# Evidence and implications of pre‐existing humoral cross‐reactive immunity to SARS‐CoV‐2

**DOI:** 10.1002/iid3.367

**Published:** 2020-12-15

**Authors:** Amandine Mveang Nzoghe, Paulin N. Essone, Marielle Leboueny, Anicet Christel Maloupazoa Siawaya, Eliode Cyrien Bongho, Ofilia Mvoundza Ndjindji, Rotimi Myrabelle Avome Houechenou, Selidji Todagbe Agnandji, Joel Fleury Djoba Siawaya

**Affiliations:** ^1^ Unité de Recherche et Diagnostics Spécialisé, Service Laboratoire, CHU‐Mère‐Enfant Fondation Jeanne EBORI Libreville Gabon; ^2^ Center de Recherches Médicales de Lambaréné Lambaréné Gabon; ^3^ Institute of Tropical Medicine and German Center for Infection Research University of Tübingen Tübingen Germany

## Abstract

**Background:**

Severe acute respiratory syndrome coronavirus 2 (SARS‐CoV‐2) has emerged throughout the world. Building knowledge around Covid‐19 is crucial to devise facts based approaches to respond efficiently against this pandemic.

**Aim:**

We aimed to investigate pre‐existing humoral cross‐reactive immunity to SARS‐CoV‐2.

**Method:**

We have tested the reactivity against SARS‐CoV‐2 nucleocapsid (N) antigen of sera collected from healthy healthcare volunteers in 2014. We assessed immunoglobulins reactive against SARS‐CoV‐2 N‐antigen using a well‐validated serological platform; Elecsys assay.

**Results:**

Sera from 32 subjects (out of 135 [23.7%]) were reactive to SARS‐CoV‐2 N‐antigen, suggesting the presence of anti‐SARS‐CoV‐2 N‐antigen antibodies.

**Conclusion:**

Although the clinical relevance of the observed reactivity can only be speculated and needs to be investigated, the implication of this finding for coronavirus disease 2019 seroepidemiological survey and vaccines' clinical trials is critical.

## Graphical abstract

This is the first report clearly showing a cross‐reactive humoral response against severe acute respiratory syndrome coronavirus 2 (SARS‐CoV‐2). Our data is adding to the accumulating data that point toward the existence of cross‐reactive immunity to SARS‐CoV‐2, as it showed that years before the coronavirus disease 2019 pandemic people had cross‐reactive antibodies against SARS‐CoV‐2.

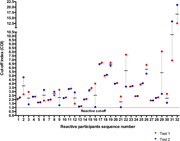

## 1 INTRODUCTION

The world is facing the most challenging health crisis of our time with the ongoing severe acute respiratory syndrome coronavirus 2 (SARS‐CoV‐2). Morbidity and mortality of this pandemic are not evenly distributed. A number of factors including pre‐existing immunity could explain the low mortality observed in some countries. In Gabon, from March (first case detected) to October 2020 more than 200,000 subjects have been screened for SARS‐CoV‐2 infection with a prevalence of 4.4% and a death rate among detected cases of 0.6% (https://africacdc.org/covid-19/). With median age around 20 years old and more than 60% of the population under 25 years old, Gabon is a country with a young population (IndexMundi). The youth of the population may only partially explain the very low mortality rate observed. Sette et al.,^1^ have illustrated a memory response to SARS‐CoV‐2 antigen from lymphocytes collected prior SARS‐CoV‐2 pandemic. We believe that the diversity of pathogens in Africa increases the population probability to be exposed to cross‐protective epitopes. The prevalence of the pre‐existing immunity in healthcare workers continuously in contact with different pathogens is unknown. The present study investigated circulating cross‐reactive antibodies against SARS‐CoV‐2 in healthcare workers.

## 2 METHODS

In this study, we selected 135 sera from healthy subjects, collected five (5) years before the first case of coronavirus disease 2019 (COVID‐19) in Gabon. The participants were initially screened for latent TB infection (QuantiFERON‐TB Gold), HIV, HVB (VIKIA HBs Ag bioMerieux), HCV (VIKIA anti HCV, bioMerieux) and syphilis. Clinical histories including Bacillus Calmette–Guérin vaccination and chronic diseases were recorded from all subjects.^2^

Using the Elecsys Anti‐SARS‐CoV‐2 immunoassay (Roche Diagnostics France) for the qualitative detection of antibodies reactive against SARS‐CoV‐2, we screened selected sera for pre‐existing humoral cross‐reactive immunity to SARS‐CoV‐2. The tests were performed following the manufacturer's instructions. The Elecsys Anti‐SARS‐CoV‐2 immunoassay detects predominantly immunoglobulin G (IgG), but also immunoglobulin A and immunoglobulin M. Sera showing an index (cutoff index [COI]) ≥ 1.0 was said to be reactive illustrating the presence of antibodies reactive against SARS‐CoV‐2 in the sera. Reactive samples were re‐assayed in an independent experiment for confirmation. We also screened all reactive samples for cytomegalovirus (CMV) and herpes simplex virus‐1/2 antibodies (IgM and IgG). All participants signed written informed consent before enrollment.

## 3 RESULTS AND DISCUSSION

We screened 135 sera that we collected from healthy subject 5 years before the COVID‐19 pandemic) for their reactivity to SARS‐CoV‐2 nucleocapsid (N) antigen. The sera were from 88 females (65.2%) and 47 males (34.8%), aged between 14 and 80 years old. The median age was 38 years (clinical and anthropomorphic data of all study subjects are in the supplementary file).

**Figure 1:**
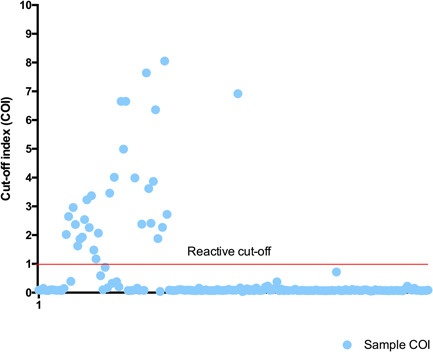
Cutoff index (COI) of sera collected from the community years before the coronavirus disease 2019 (COVID‐19) pandemic. A COI ≥ 1.0 indicates a reactive sample (positive for anti‐severe acute respiratory syndrome coronavirus 2 [SARS‐CoV‐2] antibodies)

**Figure 2:**
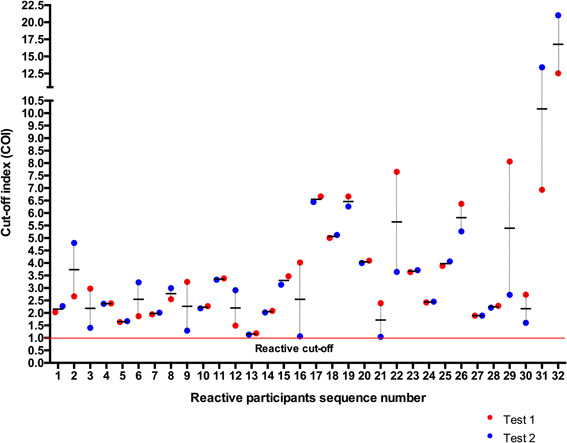
COI of reactive samples collected years before the COVID‐19 pandemic. The figure shows the results of two independent tests. COI, cutoff index; COVID‐19, coronavirus disease 2019

Overall, 23.7% (32 out of 135) of the tested sera were reactive to the SARS‐CoV‐2 recombinant nucleocapsid (N) antigen. Clinical and anthropomorphic data of subjects whom sera were reactive to the SARS‐CoV‐2 are confined in Table 1. Figure 1 shows the distribution of the samples COI. Most reactive samples had COI between two and five (84% of reactive samples) indicating a strong antigens recognition in these samples. Only five participants (15.6%) had COI less than two (but above one). Antigen recognition was particularly high in six participants with COI above five. The observed COI were confirmed in a second experiment (Figure 2). Four of the six participant with COI above five confirmed their strong reactivity. Cross‐reactivity was confirmed in all samples in the second experiment indicating a robust reaction. This is the first report explicitly showing a cross‐reactive humoral response against SARS‐CoV‐2 in Africa particularly in healthcare workers. Our data is adding to the accumulating data that point toward the existence of cross‐reactive immunity to SARS‐CoV‐2.^1^ It showed that years before the COVID‐19 pandemic people had cross‐reactive antibodies against SARS‐CoV‐2. This observation not only supports the existence of a pre‐existing cross immunity in the African population.^3^, ^4^, ^5^

**Table 1:** Clinical and anthropomorphic data of subjects whom sera were reactive to the anti‐SARS‐CoV‐2 immunoassay

**Given ID**	**Age**	**Sex**	**BMI**	**ANTI‐SARS‐CoV‐2 Ig**	**CMV (IgM/IgG)**	**HSV‐1&2 (IgM/IgG)**	**Latent TB**	**HIV‐1 & 2‐antibodies**	**HBV**	**HCV‐antibodies**	**Syphilis**	**BCG‐vaccinated**	**Chronic disease**
ID‐036	32	F	25.6	Positive	Negative	Negative	Negative	Negative	Negative	Negative	Negative	Yes	No
ID‐037	38	F	23.39	Positive	IgG positive	IgG positive	Negative	Negative	Negative	Negative	Negative	Yes	Arthritis
ID‐039	42	M	24.49	Positive	Negative	Negative	Positive	Negative	Negative	Negative	Negative	Yes	No
ID‐40	31	M	23.72	Positive	Negative	Negative	Negative	Negative	Negative	Negative	Negative	No	No
ID‐041	32	F	30.1	Positive	IgG positive	Negative	Negative	Negative	Negative	Negative	Negative	Yes	No
ID‐042	38	F	20.3	Positive	IgG positive	Negative	Negative	Negative	Negative	Negative	Negative	Yes	No
ID‐043	55	F	42.52	Positive	IgG positive	Negative	Negative	Negative	Negative	Negative	Negative	No	No
ID‐044	40	F	25.39	Positive	Negative	Negative	Negative	Negative	Negative	Negative	Negative	Yes	No
ID‐045	45	F	25.39	Positive	Negative	IgG positive	Negative	Negative	Negative	Negative	Negative	Yes	No
ID‐046	58	F	28.13	Positive	Negative	Negative	Negative	Negative	Negative	Negative	Negative	Yes	No
ID‐047	50	F	35.06	Positive	Negative	Negative	Negative	Negative	Negative	Negative	Negative	Yes	No
ID‐048	48	F	35.38	Positive	Negative	Negative	Positive	Negative	Negative	Negative	Negative	No	No
ID‐050	51	F	40.31	Positive	Negative	Negative	Negative	Negative	Negative	Negative	Negative	Yes	No
ID‐051	32	F	26.23	Positive	IgG positive	Negative	Negative	Negative	Negative	Negative	Negative	Yes	No
ID‐056	42	F	21.78	Positive	Negative	Negative	Negative	Negative	Negative	Negative	Negative	Yes	No
ID‐060	35	M	23.94	Positive	Negative	Negative	Positive	Negative	Negative	Negative	Negative	Yes	No
ID‐063	38	M	43.56	Positive	Negative	Negative	Negative	Negative	Negative	Negative	Negative	Yes	No
ID‐064	28	F	30.47	Positive	IgG positive	Negative	Negative	Negative	Negative	Negative	Negative	No	No
ID‐065	55	F	32.46	Positive	Negative	Negative	Negative	Negative	Negative	Negative	Negative	Yes	Hypertension
ID‐069	52	F	35.67	Positive	Negative	Negative	Positive	Negative	Negative	Negative	Negative	Yes	No
ID‐072	25	F	22.27	Positive	IgG positive	Negative	Negative	Negative	Negative	Negative	Negative	No	No
ID‐074	65	F	39.45	Positive	Negative	Negative	Negative	Negative	Negative	Negative	Negative	Yes	Vascular insufficiency
ID‐075	33	F	NI	Positive	Negative	Negative	Negative	Negative	Negative	Negative	Negative	Yes	No
ID‐078	40	M	30.12	Positive	Negative	Negative	Positive	Negative	Negative	Negative	Negative	Yes	No
ID‐079	38	F	27.73	Positive	Negative	Negative	Negative	Negative	Negative	Negative	Negative	Yes	Hypertension
ID‐080	23	F	44.14	Positive	Negative	Negative	Negative	Negative	Negative	Negative	Negative	Yes	No
ID‐081	23	F	22.58	Positive	Negative	Negative	Positive	Negative	Negative	Negative	Negative	Yes	No
ID‐083	32	F	26.67	Positive	Negative	Negative	Negative	Negative	Negative	Negative	Negative	NI	No
ID‐84	38	F	26.99	Positive	Negative	Negative	Negative	Negative	Negative	Negative	Negative	NI	No
ID‐087	27	F	32.05	Positive	IgG positive	Negative	Negative	Negative	Negative	Negative	Negative	NI	No
ID‐CC50	28	F	23.34	Positive	Negative	Negative	Positive	Negative	Negative	Negative	Negative	Yes	No
ID‐CC63	36	F	NI	Positive	Negative	Negative	Positive	Negative	Negative	Negative	Negative	Yes	No

Abbreviations: BCG, Bacillus Calmette–Guérin; BMI, body mass index; CMV, cytomegalovirus; HBV, hepatitis B virus; HCV, hepatitis C virus; HIV, human immunodefeciency virus; HSV, herpes simplex virus; IgG, immunoglobulin; IgM, immunoglobulin M; SARS‐CoV‐2, severe acute respiratory syndrome coronavirus 2.

One could attribute the occurrence of the SARS‐CoV‐2 cross‐reactive humoral response to the similarity between coronaviruses N‐proteins. However, a study showed no cross‐reactivity between anti‐SARS‐CoV‐2 antibodies and the N protein from common cold coronaviruses.^6^ The Elecsys Anti‐SARS‐CoV‐2 assay used to identify SARS‐CoV‐2 reactive antibodies has been evaluated in several independent studies and is considered as one of the most accurate COVID‐19 serological test.^3^, ^5^ Although this assay is highly sensitive (above 98%) and specific (above 95%), according to the manufacturer, very few sera from acute CMV and Epstein–Barr virus (EBV) infections were reactive to the Elecsys Anti‐SARS‐CoV‐2 assay.^7^ This suggests cross‐reactive antibodies in the sera of selected acute CMV and EBV patients. We would argue that the cross‐reactivity observed by the manufacturer for CMV and EBV may be due pre‐existing cross‐reactive antibodies in these samples and not to the similarity of epitopes between CMV, EBV and SARS‐CoV‐2. Because, if the similarity of epitopes was the cause of cross‐reactivity, the percentage of CMV and EBV samples positive on the Elecsys Anti‐SARS‐CoV‐2 immunoassay would have been much higher.

In our study, although the sera used were from healthy subjects with no signs of infection, 8 out of the 32 SARS‐CoV‐2 reactive sera (25%) were positives for anti‐CMV IgG. One could be tempted to link the observed cross reactivity to selected anti‐CMV IgG antibodies. We believe that any infection that leads to the production of antibodies that cross react with SARS‐CoV‐2 antigens need to be investigated further to substantiate or confute the development of a cross protective immunity. If the jury is still out regarding the origin or the source of pre‐existing cross‐reactive antibodies to SARS‐CoV‐2, a high correlation between the presence of antibodies targeting against the N‐protein and SARS‐CoV‐2 neutralizing antibodies have been demonstrated.^8^, ^9^ Therefore, cross‐reactive antibodies to SARS‐CoV‐2 may confer partial immunity that could be beneficial, as there is substantial evidence showing that pre‐existing cross‐reactive immunity can be beneficial.^10^, ^11^

Pre‐existing cross‐reactive humoral immunity in a segment of the population may have its drawbacks. Sero‐epidemiological surveys provide data that would make it possible to estimate the penetration of the virus into the population (infection attack rates) and to assess herd immunity. Here, pre‐existing cross‐reactive antibodies to SARS‐CoV‐2 N‐protein would lead to misinterpretation of the epidemiological situation. This finding has also an implication for COVID‐19 vaccine clinical trials. In the present's light, COVID‐19 vaccines clinical trials volunteers will have to undergo screening for pre‐existing cross‐immunity to SARS‐CoV‐2; to guaranty the integrity of the trials. There is a possibility that pre‐existing antibodies against SARS‐CoV‐2 might be detrimental.^12^, ^13^ The original antigenic sin, which describes the phenomenon whereby the development of immunity against antigen, is negatively shaped by the first exposure to a related antigen, is an example. It is clear that pre‐existing immune reactivity to SARS‐CoV‐2 is present in part of the population of all regions. The beneficial or detrimental effects of this pre‐existing immune reactivity to SARS‐CoV‐2 need to be investigated further.

## 4 CONCLUSION

Cross‐reactive antibodies to SARS‐CoV‐2 are present in SARS‐CoV‐2 nonexposed people. Are these antibodies protectives? What infections may lead to cross protective immunity to SARS‐CoV‐2? Those questions could represent the starting point of perspective studies.

## CONFLICT OF INTERESTS

The authors declare that there are no conflict of interests.

## AUTHOR CONTRIBUTIONS

Joel Fleury Djoba Siawaya is the principal investigator who conceived, designed the study, analyzed the data, and wrote the paper. Amandine Mveang Nzoghe and Paulin N Essone participated in study design samples processing and experiments. Marielle Leboueny, Anicet Christel Maloupazoa Siawaya, Ofilia Mvoundza Ndjindji, and Rotimi Myrabelle Avome Houechenou helped in the recruitment of participants, acquisition the samples, experiments, and the study organization. Selidji Todagbe Agnandji study critical analysis and writing.

## Supporting information

Supporting information.

## Data Availability

The dataset on which this paper is based (documentation, raw data file, and methods) used to support this study is available from (Prof Joel Fleury DJOBA SIAWAYA: joel.djoba@gmail.com).
